# Linear Energy Transfer-Dependent Change in Rice Gene Expression Profile after Heavy-Ion Beam Irradiation

**DOI:** 10.1371/journal.pone.0160061

**Published:** 2016-07-27

**Authors:** Kotaro Ishii, Yusuke Kazama, Ryouhei Morita, Tomonari Hirano, Tokihiro Ikeda, Sachiko Usuda, Yoriko Hayashi, Sumie Ohbu, Ritsuko Motoyama, Yoshiaki Nagamura, Tomoko Abe

**Affiliations:** 1 RIKEN Nishina Center, Wako, Saitama, Japan; 2 Faculty of Agriculture, University of Miyazaki, Miyazaki, Japan; 3 Agrogenomics Research Center, National Institute of Agrobiological Sciences, Tsukuba, Ibaraki, Japan; Chiba University, JAPAN

## Abstract

A heavy-ion beam has been recognized as an effective mutagen for plant breeding and applied to the many kinds of crops including rice. In contrast with X-ray or γ-ray, the heavy-ion beam is characterized by a high linear energy transfer (LET). LET is an important factor affecting several aspects of the irradiation effect, e.g. cell survival and mutation frequency, making the heavy-ion beam an effective mutagen. To study the mechanisms behind LET-dependent effects, expression profiling was performed after heavy-ion beam irradiation of imbibed rice seeds. Array-based experiments at three time points (0.5, 1, 2 h after the irradiation) revealed that the number of up- or down-regulated genes was highest 2 h after irradiation. Array-based experiments with four different LETs at 2 h after irradiation identified LET-independent regulated genes that were up/down-regulated regardless of the value of LET; LET–dependent regulated genes, whose expression level increased with the rise of LET value, were also identified. Gene ontology (GO) analysis of LET-independent up-regulated genes showed that some GO terms were commonly enriched, both 2 hours and 3 weeks after irradiation. GO terms enriched in LET-dependent regulated genes implied that some factor regulates genes that have kinase activity or DNA-binding activity in cooperation with the *ATM* gene. Of the LET-dependent up-regulated genes, *OsPARP3* and *OsPCNA* were identified, which are involved in DNA repair pathways. This indicates that the Ku-independent alternative non-homologous end-joining pathway may contribute to repairing complex DNA legions induced by high-LET irradiation. These findings may clarify various LET-dependent responses in rice.

## Introduction

Rice is one of the most important cereal grains consumed as a staple by over half of the world’s population. Many rice varieties have been produced through mutation breeding. For example, among the 3226 mutant varieties listed on the FAO/IAEA Database (http://mvgs.iaea.org/AboutMutantVarities.aspx), 814 (approximately 25%) are of rice (as of Feb. 2016).

The heavy-ion beam, a type of ionizing radiation, is an effective mutagen that has been used in genetics and mutation breeding in plants and microorganisms. The heavy-ion beam has advantages for mutation breeding, such as high mutation frequencies, wide phenotypic spectrum, and a minimum effect on the non-targeted trait [1, 2]. Hence, mutants induced by heavy-ion irradiation become new cultivars. In Japan, over 40 cultivars have been produced using heavy-ion irradiation. One successful example is a reduced cadmium uptake mutant bred from a major Japanese rice cultivar, Koshihikari [3].

The heavy-ion beam deposits its energy into a highly dense region along the particle path, whereas X-rays or γ-rays deposit their energy separately. Thus, the heavy-ion beam applies a large amount of energy in a small area, when scaled along the direction of the beam. The deposited energy per unit pass length is designated as linear energy transfer (LET). Thus, the heavy-ion beam is called high-LET radiation. The LET of γ-rays is 0.2 keV μm^-1^, while that of heavy-ion beams varies depending on the valency or velocity of the ions.

The LET affects many aspects of the irradiated cells, including cell-death, and mutation induction. In animal cells, about 100 keV μm^-1^ of LET is most effective for both cell-death and mutation induction [4]. For dry seed irradiation of the model plant, *Arabidopsis thaliana*, the most effective LETs on killing and mutation induction are around 290–400 keV μm^-1^ [5–7] and 30.0 keV μm^-1^ [5, 8, 9], respectively.

The effect of LET on inducing double strand break (DSBs) has been simulated using Monte Carlo calculations [10]. The effect on the deletion induction increases up to an LET value of around 100 keV μm^-1^. In contrast, the mutation frequency increases abruptly and peaks at a particular LET value [5, 8]. Low mutation frequency produced by higher-LET irradiation is likely due to fewer irradiated particles. Because the dose is proportional to the product of the LET multiplied by the number of ion particles, the number of particles in higher-LET radiation is lower than that of lower-LET at the same dose. However, the mechanism between LET and the corresponding mutation frequency is still unknown.

One possibility in discovering this mechanism is to investigate the gene expression profile. In plants, the change in the gene expression profile after irradiation of ionizing radiation has been studied [11]. In rice, one study investigated the dose-dependent change of the gene expression profile after irradiation of a carbon-ion beam with a microarray experiment and revealed that the number of genes whose expression level changed was dose-dependent immediately after irradiation; the genes involved in signal transduction showed the highest change in their expression level [12]. Other microarray studies investigating gene expression responses to γ-ray irradiation and six other abiotic stresses revealed these genes to be up-regulated specifically by the γ-ray irradiation, and many of these genes functioned in oxidative stress response [13]. These studies successfully identified the genes responding to ionizing radiation using microarray analysis. However, the LET effect on the gene expression profile has not been investigated.

In this study, we focused on the effect of LET on gene expression profiles at the same dose by using the well-developed rice array platform [14]. This project aims to build a molecular framework for future research on the relationship between LET and biological response.

## Materials and Methods

### Plant materials and heavy-ion beam irradiation

*Oryza sativa* L. ‘Nipponbare’ seeds were imbibed for 3 days. ^12^C^6+^ ions and ^20^Ne^10+^ ions were accelerated up to 135 MeV nucleon^-1^ using an azimuthally varying field cyclotron and RIKEN Ring Cyclotron at the RIKEN RI Beam Factory. The seeds were arranged in a single layer in a plastic petri dish. A part of the imbibed seeds were used as un-irradiated controls. Then the remaining seeds were irradiated with 22.5 or 50 keV μm^-1^ C-ion or with 63 or 80 keV μm^-1^ Ne-ion at a dose of 15 Gy. The LETs were calculated on the surface of the seeds. The numbers of particles hitting an area of 10 μm^2^ on the surface of the seeds were calculated as previously described [8], to be 416, 187, 149, and 117 for 22.5-,50-, 63-, and 80keV μm^-1^ beams, respectively.

### Microarray analysis

Embryos sampled from both the irradiated and un-irradiated seeds at 0.5, 1, and 2 hours after heavy-ion beam irradiation were immediately frozen in liquid nitrogen. Total RNA was isolated using the RNeasy Plant Mini Kit (Qiagen, Tokyo, Japan) and labelled using the Quick Amp Labeling Kit (Agilent Technologies, Tokyo, Japan) according to manufacturers' instructions. Gene expression profiles were generated using a rice 4×44K microarray RAP-DB (Agilent Technologies; G2519F#15241, Tokyo, Japan) according to the manufacturer instructions. Each experiment was performed three times. Gene expression profiles were analysed on a Subio Platform (ver. 1.15; Subio, Kagoshima, Japan). Expression data with a raw intensity less than 30, or with the flag ‘gIsWellAboveBG’ set at 0 for all samples in each analysis were excluded. The data was normalized using the ‘Profiling’ pre-set scenario, which consists of four processes: the ‘Low Signal Cutoff’ with the ‘Cutoff’ value of 1.0 and the ‘Replace’ option, the ‘Log Transformation’ with the ‘Base 2’ option, the ‘Global Normalization’ with the ‘Percentile’ value of 75, and the ‘Centering’ with the ‘Mean’ option. Expression data of three replicates (samples irradiated with the same LET plus un-irradiated control and sampled at the same time) were grouped in one ‘Sample Group’. Expression data of two sample groups were compared using a ‘Compare 2 Groups’ plug-in. Genes showing more than two-fold change (FC > 2 or 0.5) using the Student’s t-test (p < 0.05) in expression level were classified as significantly up- or down-regulated genes, respectively. NCBI GEO accession numbers is GSE78998 (subseries GSE78997 is for analysis of temporal change in gene expression profile; GSE78994 is for analysis of difference in gene expression profile depending on LET).

### Singular Enrichment Analysis (SEA)

Gene locus names used in the microarray originate from the Rice Annotation Project Database, and those in a gene group used for SEA were converted to the MSU Rice Genome Annotation Project on the OryzaExpress web-based software (http://bioinf.mind.meiji.ac.jp/OryzaExpress/ID_converter.php). Genes not having the MUS locus name were excluded from the analysis. SEA was conducted using the agriGO web-based software (http://bioinfo.cau.edu.cn/agriGO/analysis.php) [15]. It was executed with the ‘Select the species’ option set to ‘*Oryza sativa*’, the ‘Select reference’ option set to ‘Rice TIGR locus’, and other ‘Advanced options’ kept at default values.

## Results

### Temporal change in gene expression profile

We treated *O*. *sativa* imbibed seeds with a carbon-ion beam at two different LETs (22.5 and 50 keV μm^-1^), then sampled the seeds 0.5, 1, and 2 hours after irradiation and conducted microarray analysis. We counted the number of significantly up- or down-regulated genes (FC > 2 or < 0.5, and p < 0.05) (Table 1). Of the three time points, the number of genes altering their expression levels was the lowest and highest 0.5 and 2 hours after irradiation with both LETs, respectively.

**Table 1:** Number of genes showing significant change in expression level after carbon-ion irradiation.

LET (keV μm^-1^)		0.5 h^a^	1 h^a^	2 h^a^
22.5	Up-regulated	113 (131)	207 (242)	593 (730)
	Down-regulated	142 (158)	226 (286)	628 (804)
50	Up-regulated	61 (74)	335 (407)	1082 (1355)
	Down-regulated	75 (82)	318 (380)	787 (952)

Figures in parentheses indicate number of probes.

^a^Hour(s) after irradiation.

Ionizing radiation causes DSBs to some extent, which results in mutations if they are not correctly repaired. Irradiation of *A*. *thaliana* with 25 Gy of γ-ray resulted in DSBs as detected by immunostaining γ-H2AX [16]. Approximately 50% and 70% of γ-H2AX foci were removed following 30 min and 3 h, respectively, after irradiation. Thus, expression profiles of DSB-repair related genes can be used to investigate whether the profiles reflect the influence of irradiation. Of the known DNA repair genes in rice (S1 Table) [17–22], five genes involving homologous recombination (HR) and *OsMSH5* showed an increased expression level over time (Table 2). However, the *RecQ* homolog and *OsRPA70b* showed a decreased expression level over time (Table 2). A gene coding a heavy metal transport/detoxification protein (Os02g0818900) that was reported as an ionizing radiation (IR) indicator [12] also showed increased expression level at all three time points for both LETs. This was especially at 2 hours after irradiation, where the expression levels were more than 100 times higher than that of the control. These results indicate that this microarray analysis reflects the response of the gene expression profile to heavy-ion beam irradiation.

**Table 2:** Time-course of the change in expression of relevant genes.

Gene locus ID (Gene name)	Agilent feature number	LET (keV μm^-1^)	Fold change						Pathway
			0.5 h^a^		1 h^a^		2 h^a^		
Os12g0143800 (*OsDMC1A*)	635	22.5	1.08		1.50	**	1.85	**	Homologous recombination^b^
		50	1.05		1.70	**	2.32	**	
	14550	22.5	1.08		1.49	**	1.84	**	
		50	1.03		1.63	**	2.35	**	
	20114	22.5	1.08		1.53	**	1.79	**	
		50	1.01		1.66	**	2.26	**	
Os11g0146800 (*OsDMC1B*)	1628	22.5	1.22		2.10	*	3.23	**	
		50	1.15		2.32	**	3.84	**	
	12166	22.5	1.26		2.09	*	3.28	**	
		50	1.13		2.25	**	3.98	**	
Os12g0497300 (*OsRad51A2*)	1780	22.5	9.32	**	9.65	**	8.13	**	
		50	8.37	**	14.0	**	8.71	**	
	20749	22.5	7.21	**	9.35	**	8.13	**	
		50	6.06	**	13.6	**	8.98	**	
Os02g0762800 (*OsRad54*)	13523	22.5	1.58		1.88	*	2.28	**	
		50	1.38		1.82	**	2.12	**	
Os05g0512000 (*OsBRCA1*)	40839	22.5	1.62	*	2.25	**	2.67	**	
		50	1.48		2.59	**	3.31	**	
Os05g0498300 (*OsMSH5*)	40156	22.5	1.16		1.59	**	2.16	**	Mismatch repair^b^
		50	1.13		1.63	**	2.33	**	
Os11g0672700 (*RecQ* homolog)	118	22.5	1.38		1.26		0.62		RecQ helicase^b^
		50	1.19		1.05		0.38	**	
Os03g0214100 (*OsRPA70b*)	9663	22.5	1.46	*	1.05		0.42	*	Replication protein A^b^
		50	1.07		0.83		0.27	**	
	25758	22.5	1.38	*	1.06		0.44	*	
		50	1.08		0.83		0.28	**	
	35147	22.5	1.46		1.05		0.44	*	
		50	1.12		0.82		0.30	**	
Os04g0488100 (*OsRad21-2*)	8677	22.5	2.33	**	4.11	**	4.42	**	Cohesin complex
		50	2.17	**	5.06	**	5.33	**	
	30753	22.5	2.16	**	4.06	**	4.48	**	
		50	2.03	**	4.99	**	5.25	**	
Os02g0818900 (IR indicator)	33750	22.5	7.46	**	18.6	*	113	**	-
		50	5.82	**	36	**	188	**	

^a^Hour(s) after irradiation.

^b^Classification of pathway was based on Kimura et al. [18].

* p < 0.05,

** p < 0.01.

### Difference in gene expression profile in terms of LET

Under the same absorbed dose, the particle number of the heavy-ion beam is inversely proportional to the value of LET. In the irradiation of 15 Gy, particle numbers are 416, 187, 149, and 117 for the LETs of 22.5, 50, 63 and 80 keV μm^-1^, respectively. To determine the effect of LET on gene expression profiles, we irradiated a neon-ion beam on imbibed seeds at two different LETs (63 and 80 keV μm^-1^). We sampled the seeds 2 hours after irradiation, when the number of genes showing significant change in expression level would be largest (Table 1), and then conducted additional microarray hybridization for the samples irradiated with the two different LETs. Data analysis was conducted for microarray data sampled 2-hours after irradiation with 22.5, 50, 63 and 80 keV μm^-1^. With the LETs at 22.5, 50, 63, and 80 keV μm^-1^, 581, 1078, 952, and 1071 genes were up-regulated, while 648, 813, 793, and 1069 genes were down-regulated compared to the non-irradiated controls, respectively (Table 3).

**Table 3:** Number of genes showing significant changes in expression level at 2 h after irradiation.

Ion species	LET (keV μm^-1^)	Up-regulated	Down-regulated
Carbon	22.5	581 (716)	648 (825)
	50	1078 (1348)	813 (982)
Neon	63	952 (1188)	793 (1002)
	80	1071 (1347)	1069 (1353)
	LET-independent regulated	379 (469)	265 (328)
	LET-dependent regulated	628 (781)	594 (694)

Figures in parentheses indicate number of probes.

Data are lacking on the effect of LET value on gene expression levels, so we defined genes showing a significant change in expression level (FC > 2 or < 0.5, and p < 0.05) at an LET of 80 keV μm^-1^, but not at 22.5 keV μm^-1^, as ‘LET-dependent regulated genes’ tentatively. In total, 628 and 594 LET-dependent up- and down-regulated genes were identified, respectively (Table 3). However, the genes which were significantly up- or down-regulated (FC > 2 or < 0.5, and p < 0.05) at all LETs (22.5, 50, 63, and 80 keV μm^-1^) were defined as ‘LET-independent regulated genes’. The LET-independent up- and down-regulated genes were 379 and 265, respectively (Table 3). The indicator for ionizing radiation (Os02g0818900) and five of the DNA repair genes listed in Table 2; *OsDMC1B*, *OsRad51a2*, *OsRad54*, *OsBRCA1*, and *OsMSH5* were all LET-independent up-regulated (S2 Table).

### Gene Ontology analysis of LET-dependent/independent regulated genes

**Fig 1:**
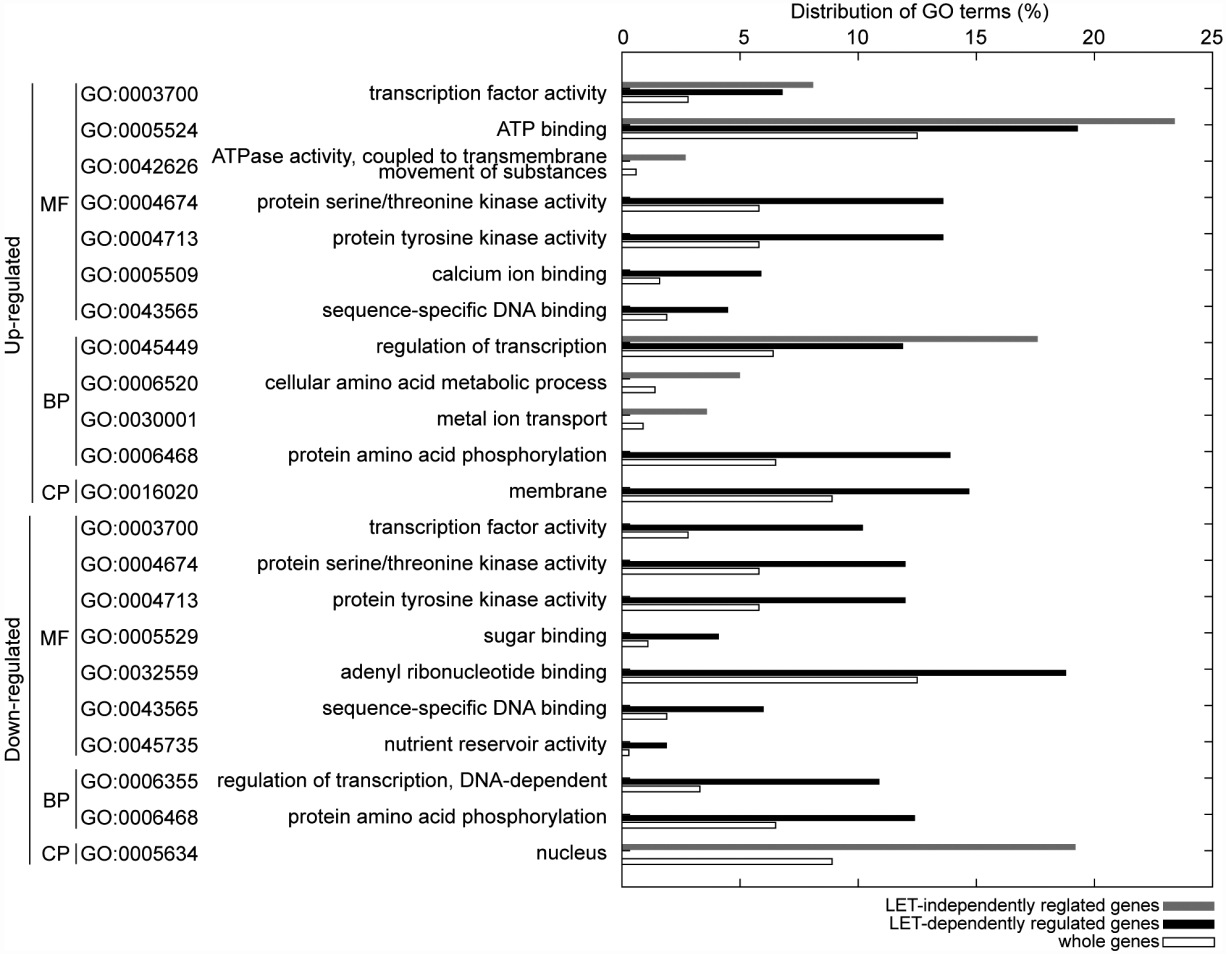
SEA analysis of LET-independent and -dependent regulated genes. GO terms in the GO graph structure which were significantly enriched (p ≤ 0.01) are listed. Horizontal axis shows distribution of GO term that is a percentage of genes having each GO term in analysed gene set. Grey, black, and white boxes indicate distribution of LET-independent regulated genes, LET-dependent regulated gene, and whole genes (rice TIGR locus).

**Fig 2:**
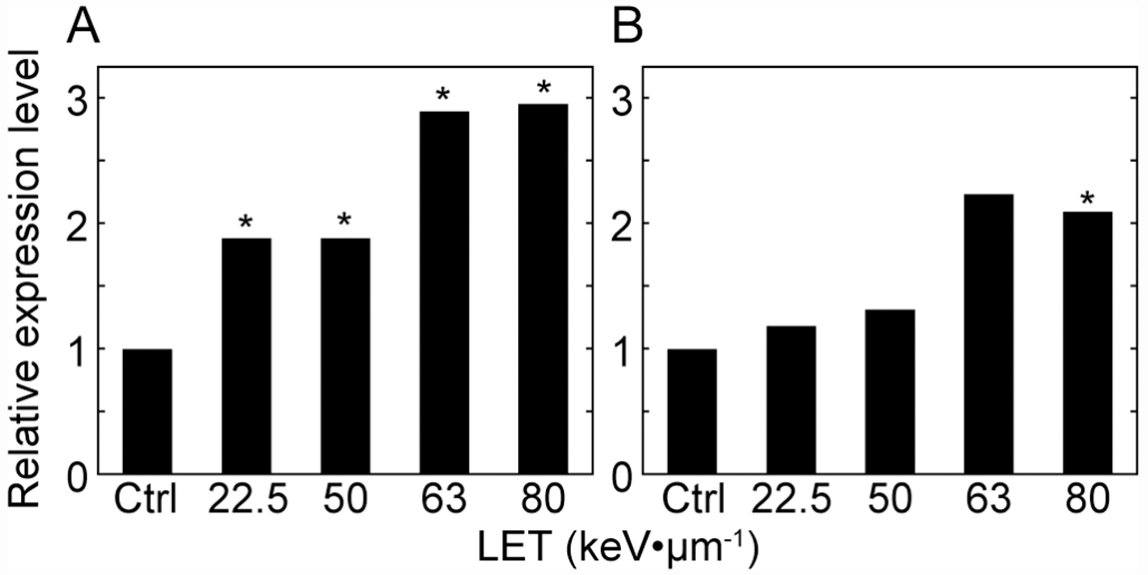
Response of *OsPCNA* and *OsPARP3* gene expressions to heavy-ion beam irradiation with different LETs. Fold changes in expression level of *OsPCNA* (A) and *OsPARP3* (B) compared to the un-irradiated control are shown. *: p < 0.05.

To characterize LET-dependent or -independent regulated genes, we conducted an SEA analysis using agriGO [15]. SEA revealed that gene ontology (GO) terms such as ‘protein serine/threonine kinase activity’, ‘protein tyrosine kinase activity’, ‘calcium ion binding’, ‘sequence-specific DNA binding’, and related terms within the molecular function (MF) ontology, as well as ‘protein amino acid phosphorylation’, and their parent terms in the biological process (BP) ontology, and ‘membrane’ in the cellular component (CP) ontology, were significantly (p ≤ 0.01) enriched in LET-dependent up-regulated genes but not in LET-independent up-regulated genes (Fig 1 and S1 and S2 Figs). This implies that a different genetic pathway is induced depending on the complexity of DNA damage (see Discussion). Additionally, *OsPCNA* and *OsPARP3* that is one of the PARP gene family, which are thought to be involved in DNA repair, were found to be LET-dependent up-regulated genes (Fig 2 and S3 Table). For the LET-dependent down-regulated genes, GO terms such as ‘transcription factor activity’, ‘protein serine/threonine kinase activity’, ‘protein tyrosine kinase activity’, ‘sugar binding’, ‘adenyl ribonucleotide binding’, ‘sequence-specific DNA binding’, ‘nutrient reservoir activity’, and their related terms, along with ‘regulation of transcription, DNA-dependent’, ‘protein amino acid phosphorylation’, and their parent terms, were enriched, but not in LET-independent down-regulated genes (Fig 1 and S3 and S4 Figs). Thus, some signal transduction pathways may be relatively inhibited in place of up-regulated pathways after high-LET irradiation (See Discussion).

For LET-independent up-regulated genes, GO terms such as ‘transcription factor activity’, ‘ATP binding’ and their parent terms in the MF ontology, as well as ‘regulation of transcription’ and its parent terms in the BP ontology, were significantly enriched in the LET-dependent up-regulated genes as well (Fig 1 and S1 and S2 Figs). The GO terms ‘ATPase activity’ and its parent terms within the MF ontology, and ‘cellular amino acid metabolic process’, ‘metal ion transport’, and their parent terms in the BP ontology, were specifically enriched in the LET-independent up-regulated genes (Fig 1 and S1 Fig). For the LET-independent down-regulated genes, the GO term ‘nucleus’ and its parent terms in the CP ontology were specifically enriched (Fig 1 and S3 Fig).

## Discussion

### Gene expression profile after heavy-ion irradiation

In this study, we characterized the gene expression profile of rice after heavy-ion beam irradiation. The number of up-/down-regulated genes was the largest at 2 hours after irradiation (Table 1). Likewise, the expression of the IR indicator gene, and some known DNA repair genes, was highly up-regulated at 2 h after irradiation (Table 2). Therefore, this time point was ideal to profile changes in gene expression after heavy-ion beam irradiation in this study. In our SEA analysis, the GO terms ‘transcription’, ‘regulation of transcription’, and ‘cellular amino acid derivative metabolic process’ within the BP ontology, and ‘transcription factor activity’ in the MF ontology, were enriched after irradiation at any LET. These GO terms were also enriched in another gene expression profiling study for rice seeds at three weeks after γ-beam irradiation [13]. One explanation is that these pathways change their expression levels 2 h after irradiation and maintain them up to 3 weeks after irradiation.

### LET-dependent regulated pathways

We identified several LET-dependent regulated pathways (Fig 1 and S2 and S4 Figs). The GO terms enriched in both LET-dependent up- and down-regulated genes include ‘protein serine/threonine kinase activity’, ‘protein tyrosine kinase activity’, ‘sequence-specific DNA binding’, and ‘protein amino acid phosphorylation’. One of the protein kinases, ATM, functions as an apical activator in response to DSB. Human ATM recognises as many as 1077 protein substrates and regulates many transcription factors, phosphorylation pathways, and kinase cascades [23]. In *A*. *thaliana*, ATM and ATR also phosphorylate many proteins in response to DNA damage [24]. It is possible that some accessorial factor that senses DSB may activate or inactivate the genes with kinase activity or DNA-binding activity in cooperation with ATM. Further analysis of mutants of each up- or down-regulated gene following high LET irradiation may show alternate DNA repairing activity. The GO term ‘nutrient reservoir activity’ was enriched in the LET-dependently down-regulated genes (S4 Fig). However, it was enriched in the up-regulated genes after low-LET γ-ray irradiation [13]. This gene expression might be suppressed if highly complex DNA lesions are induced.

### Possible role of DSB repair pathways after heavy-ion irradiation

The two major DSB repair pathways, the error-free HR and error-prone non-homologous end joining (NHEJ), are well known [25]. NHEJ is divided into the Ku-dependent canonical pathway (C-NHEJ) and the Ku-independent alternative pathway (A-NHEJ). As the expression of HR-related genes is known to be induced by DSB-inducing agents [11], the expression of HR-related genes in this study was found to be LET-independent up-regulated. The expressions of *Ku70/80*, major components of C-NHEJ, were not changed over the time period indicated in previous studies [11].

*OsRad21-2* and *OsRad51A2* showed expression changes in parallel with the time frame (Table 2). *OsRad21-2* is an orthologue of *AtRAD21*.*1* in *A*. *thaliana* and encodes a constitutive protein involved in sister chromatid cohesion during mitosis [26]. The increased expression level of *AtRAD21*.*1* after irradiation with γ-rays parallels the increased expression level of *AtRAD51* involved in HR, but the expression level does not change after irradiation with UV-B [27]. It is likely that *OsRad21-2* is also involved in DSB repair in rice.

Two DSB-repair related genes, *OsPCNA* and *OsPARP3*, were detected as LET-dependent up-regulated genes in this study (Fig 2 and S2 Fig). Although molecular functions of rice PARP family genes are largely unknown, one of the mammalian *PARP* family gene *PARP1* is involved in Ku-independent NHEJ (Alternative (A)-NHEJ) with *Xrcc1* and *DNA ligase III* in animals [16]. Conversely, the different member of the *PARP* family gene *AtPARP2* (At4g02390) is predominantly involved in A-NHEJ in *A*. *thaliana* [28, 29]. Thus, some of PARP family genes could have a role in the A-NHEJ pathway in rice. *OsPCNA* is also thought to be involved in A-NHEJ, because *OsPCNA* forms a complex with *OsXRCC1 in vivo* [30]. The contribution of A-NHEJ to DSB repair may be important in describing the LET-dependent response. In *A*. *thaliana*, radiation sensitivity of a C-NHEJ deficient mutant was more severe than that of the wild type at lower-LET irradiation than at higher-LET irradiation [31]. In animals, it has been confirmed that clustered complex DNA lesions were induced by high-LET irradiation [32–34]. These complex DNA lesions could not be repaired by the Ku-dependent pathway [35, 36]. Taken together, C-NHEJ is inefficient to repair the high-LET induced DNA lesions and A-NHEJ may contribute to the repair of these lesions. We assume that the high expression of *OsPARP3* and *OsPCNA* observed after 80-keV μm^-1^ irradiation is needed to repair the high-LET induced DNA lesions via A-NHEJ.

## Conclusions

In this study, we revealed that the change in gene expression profiles was highest at 2 h after heavy-ion beam irradiation considering three time points: 0.5, 1, and 2 h after irradiation. We also investigated gene expression profiles at 2 h after irradiation with four different values of LET. Genes which were up- or down-regulated regardless of the LET value contained eight DNA-repair related genes and one known ionizing radiation indicator gene, suggesting that expression profiles successfully reflected a response to the irradiation. LET-dependent up-regulated genes included ones having protein kinase, DNA binding, or calcium binding activities. Two DNA repair genes, *OsPARP3* and *OsPCNA*, were also included in the LET-dependent up-regulated genes. *OsPARP3* and *OsPCNA* are likely to be involved in the A-NHEJ pathway. These finding will help clarify various LET-dependent responses in rice.

## Supporting Information

S1 Fig SEA analysis of LET-independent up-regulated genes. Significantly enriched GO terms (p ≤ 0.01) are shown in coloured boxes. The level increases with increasing significance. The top lines in boxes indicate GO identifiers and p-values are in parentheses. GO terms are shown in the following line. The bottom lines in the coloured boxes contain two fractions; the numerators of the first fractions indicate the number of genes having an associated GO term in 353 LET-dependent up-regulated genes, and the numerators of the second fraction indicate the number of genes having an associated GO term in 24460 rice genes. Arrows indicate relationships among GO terms: black, red, and green arrows indicate ‘is_a’, ‘positive_regulate’, and ‘negative_regulate’ relationships, respectively. Long and short dashed lines indicate ‘two significant nodes’ and ‘one significant node’ relationships, respectively. (A) Analysis of MF ontology. (B) That of BP ontology. (C) That of CP ontology.
(PDF)

S2 Fig SEA analysis of LET-dependent up-regulated genes. GO terms and their relationships are shown in the same manner as in S1 Fig, except the first numerators of the first fractions in the bottom lines in the coloured boxes indicate the number of genes having an associated GO term in 266 LET-dependent down-regulated genes. (A) Analysis of MF ontology. (B) That of BP ontology.
(PDF)

S3 Fig SEA analysis of LET-independent down-regulated genes. GO terms and their relationships are shown in a same manner as S1 Fig, except the first numerators of the first fractions in the bottom lines in the coloured boxes indicate the number of genes having an associated GO term in 222 LET-independent up-regulated genes. (A) Analysis of MF ontology. (B) That of BP ontology.
(PDF)

S4 Fig SEA analysis of LET-dependent down-regulated genes. GO terms and their relationships are shown in the same manner as in S1 Fig, except the first numerators of the first fractions in the bottom lines in the coloured boxes indicate the number of genes having an associated GO term in 120 LET-independent down-regulated genes.
(PDF)

S1 Table List of DNA repair-related genes analysed in this study. (XLS)

S2 Table Change in expression of relevant LET-independent up-regulated genes. (XLS)

S3 Table Change in expression of *OsPCNA* and PARP family genes. (XLS)
