# Supplementary figures and images for: Linear Energy Transfer-Dependent Change in Rice Gene Expression Profile after Heavy-Ion Beam Irradiation
Source: PLoS One. 2016 Jul 27;11(7):e0160061. doi: 10.1371/journal.pone.0160061 (PMC4962992; doi:10.1371/journal.pone.0160061)

**A**

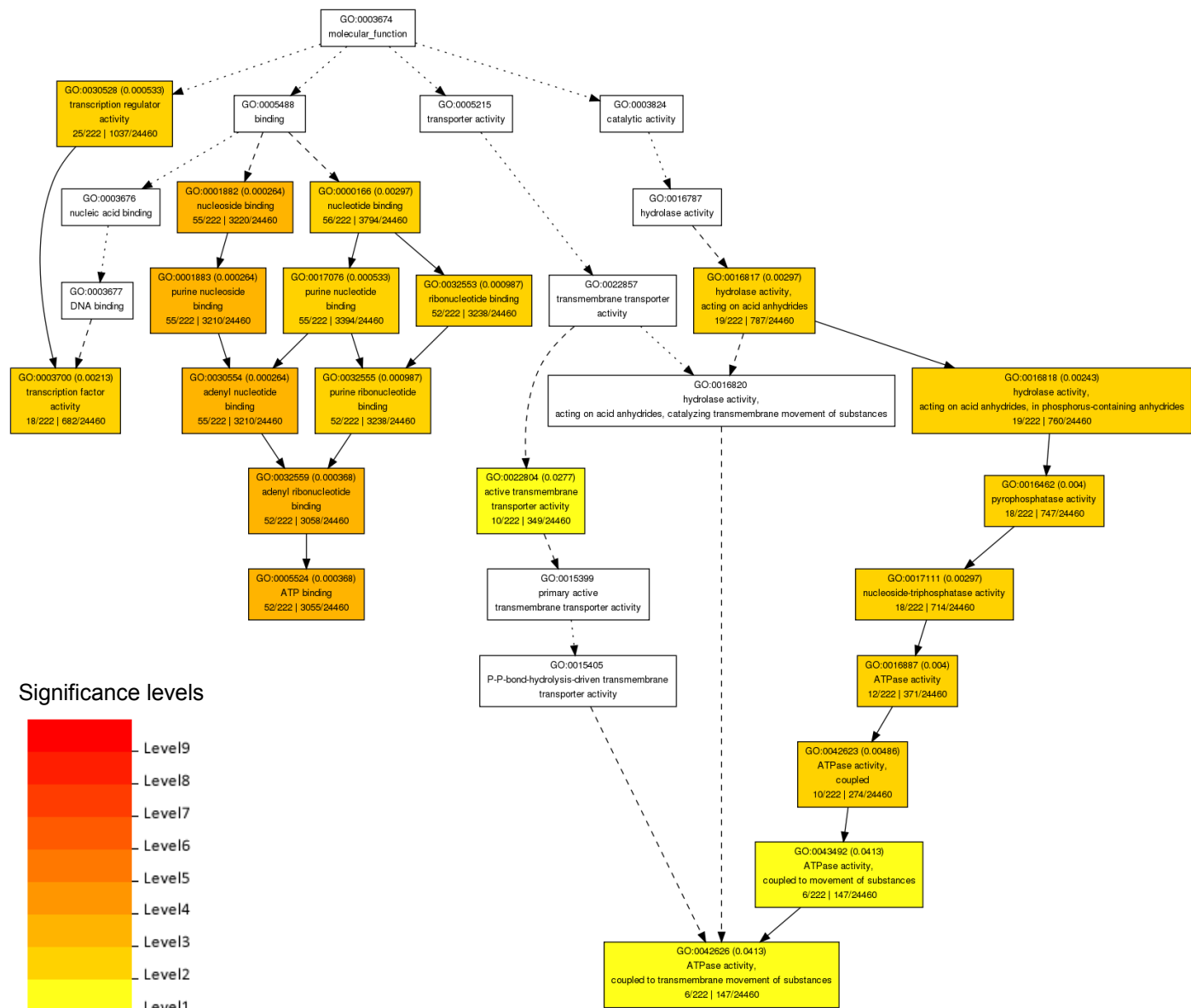

Supplement: S1 Fig — Significantly enriched GO terms (p ≤ 0.01) are shown in coloured boxes. The level increases with increasing significance. The top lines in boxes indicate GO identifiers and p-values are in parentheses. GO terms are shown in the following line. The bottom lines in the coloured boxes contain two fractions; the numerators of the first fractions indicate the number of genes having an associated GO term in 353 LET-dependent up-regulated genes, and the numerators of the second fraction indicate the number of genes having an associated GO term in 24460 rice genes. Arrows indicate relationships among GO terms: black, red, and green arrows indicate ‘is_a’, ‘positive_regulate’, and ‘negative_regulate’ relationships, respectively. Long and short dashed lines indicate ‘two significant nodes’ and ‘one significant node’ relationships, respectively. (A) Analysis of MF ontology. (B) That of BP ontology. (C) That of CP ontology. (PDF) [file pone.0160061.s001.pdf]

A

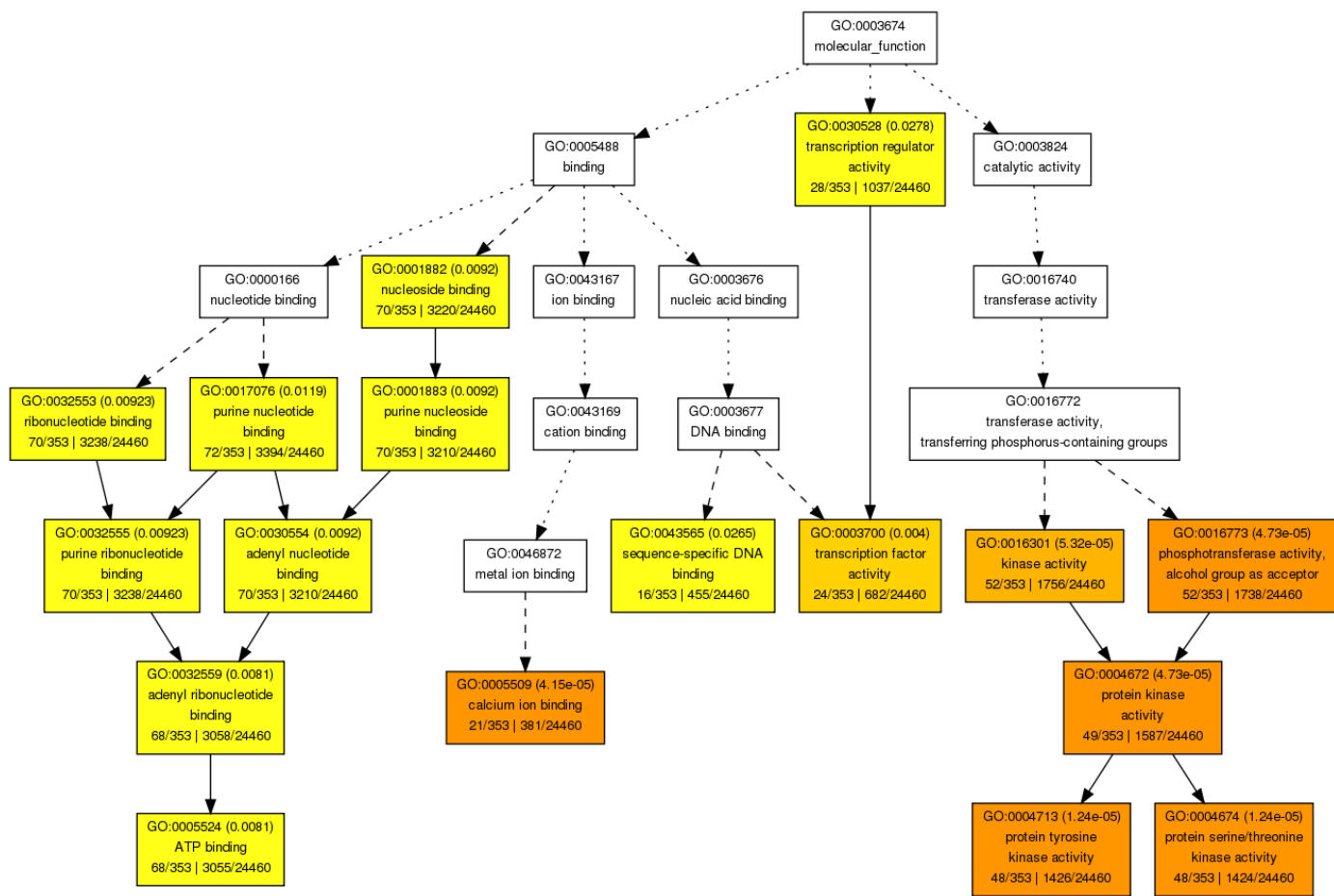

Significance levels

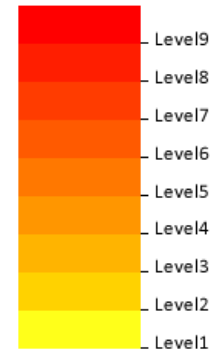

GO:0008150  
biological\_process

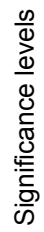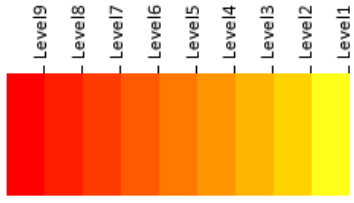

C

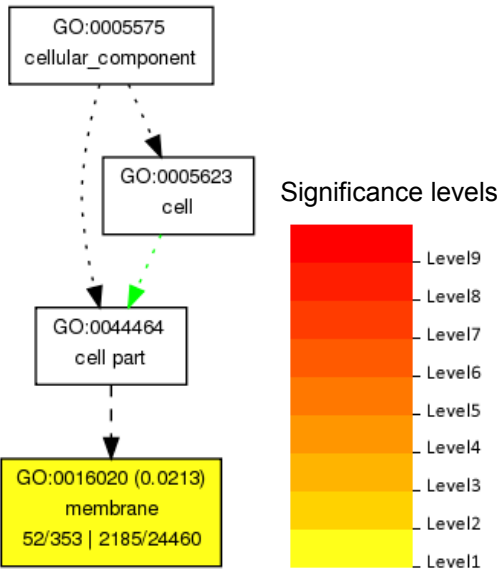

Supplement: S2 Fig — GO terms and their relationships are shown in the same manner as in S1 Fig, except the first numerators of the first fractions in the bottom lines in the coloured boxes indicate the number of genes having an associated GO term in 266 LET-dependent down-regulated genes. (A) Analysis of MF ontology. (B) That of BP ontology. (PDF) [file pone.0160061.s002.pdf]

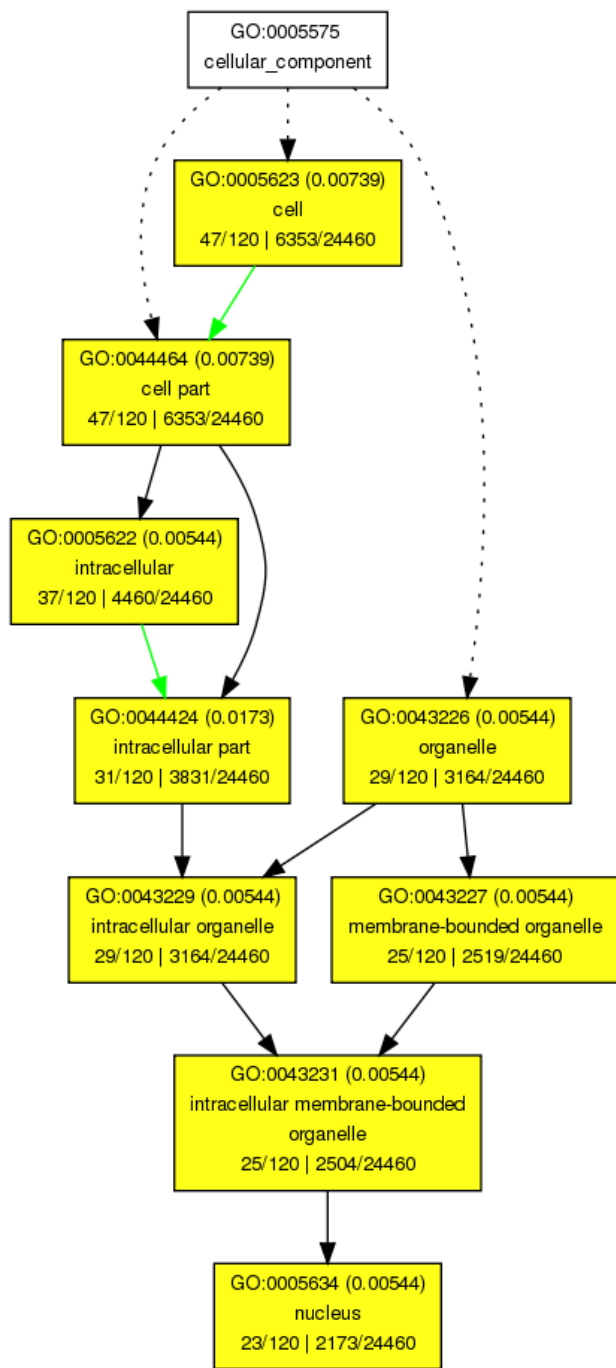

Significance levels

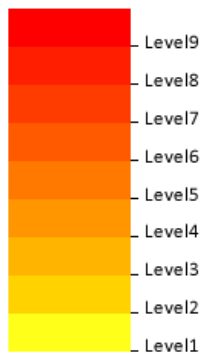

Supplement: S3 Fig — GO terms and their relationships are shown in a same manner as S1 Fig, except the first numerators of the first fractions in the bottom lines in the coloured boxes indicate the number of genes having an associated GO term in 222 LET-independent up-regulated genes. (A) Analysis of MF ontology. (B) That of BP ontology. (PDF) [file pone.0160061.s003.pdf]

A

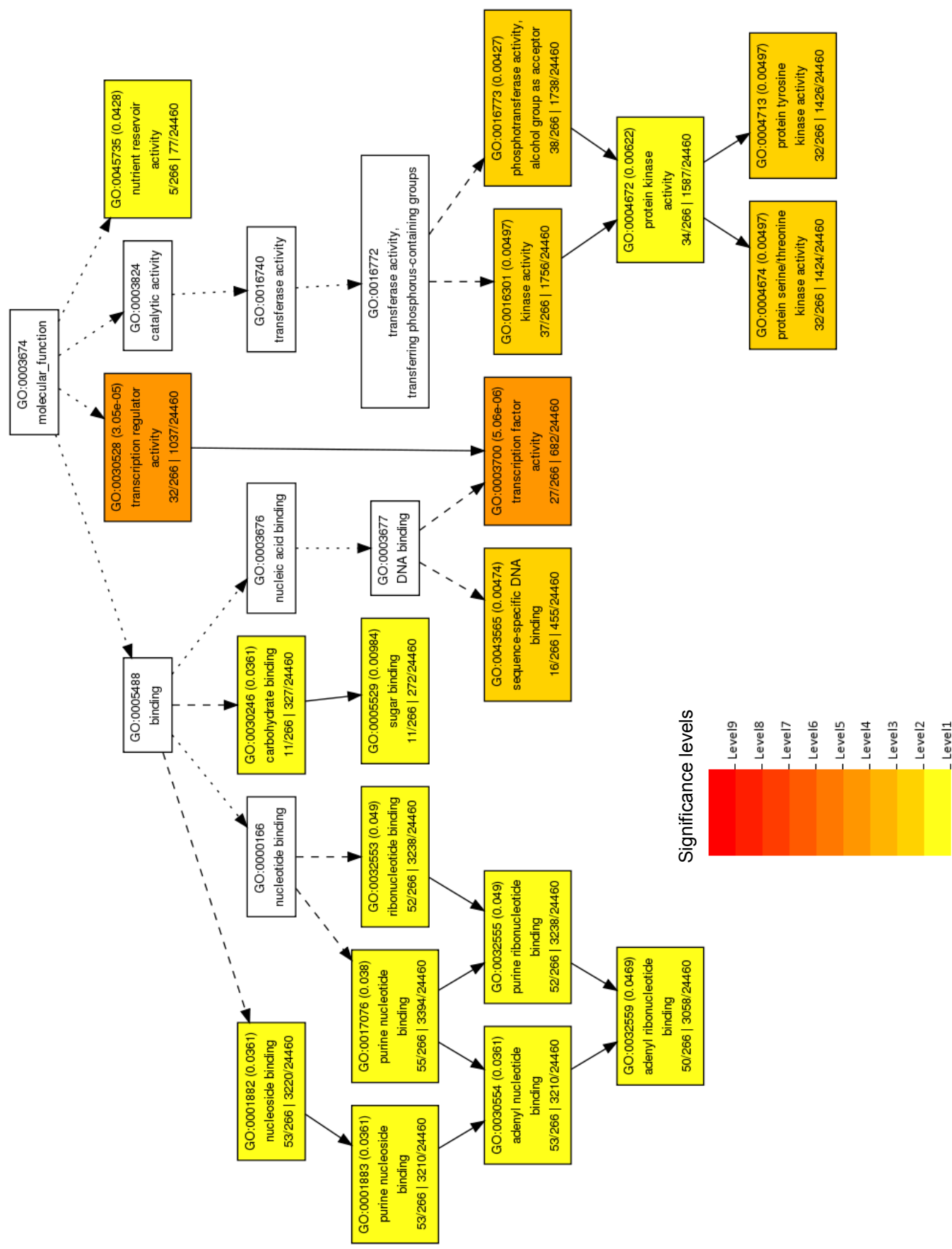

GO:0008150  
biological\_process

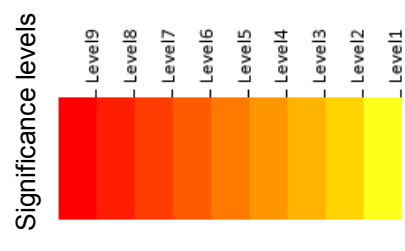

Supplement: S4 Fig — GO terms and their relationships are shown in the same manner as in S1 Fig, except the first numerators of the first fractions in the bottom lines in the coloured boxes indicate the number of genes having an associated GO term in 120 LET-independent down-regulated genes. (PDF) [file pone.0160061.s004.pdf]
